# Measuring the Surface Energy of Nanosheets by Emulsion Inversion

**DOI:** 10.1021/acs.jpcc.4c02893

**Published:** 2024-10-01

**Authors:** Anne Sehnal, Sean P. Ogilvie, Keiran Clifford, Hannah J. Wood, Aline Amorim Graf, Frank Lee, Manoj Tripathi, Peter J. Lynch, Matthew J. Large, Shayan Seyedin, Kathleen Maleski, Yury Gogotsi, Alan B. Dalton

**Affiliations:** † School of Mathematical and Physical Sciences, University of Sussex, Brighton BN1 1RH, U.K.; ‡ School of Engineering, Newcastle University, Newcastle upon Tyne NE1 7RU, U.K.; § A. J. Drexel Nanomaterials Institute, and Department of Materials Science and Engineering, Drexel University, Philadelphia, Pennsylvania 19104, United States

## Abstract

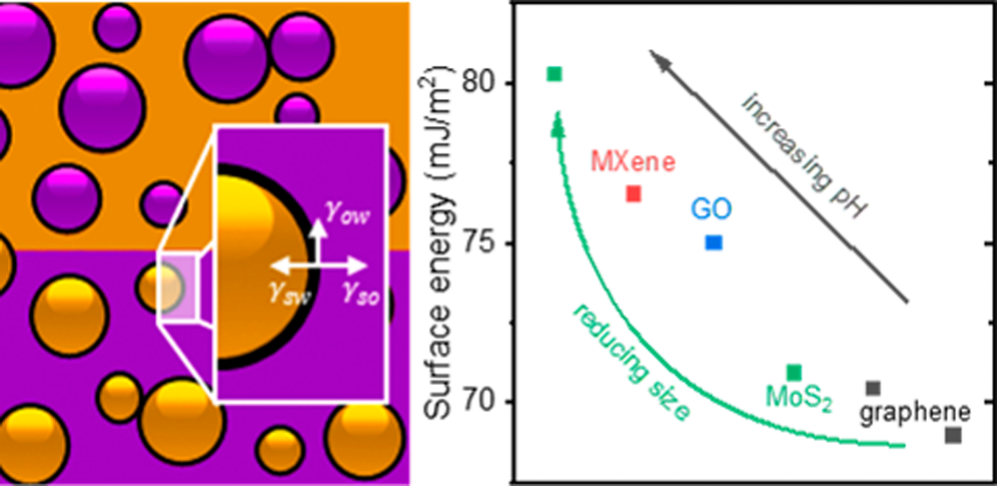

Solution-processed nanomaterials can be assembled by a range of interfacial techniques, including as stabilizers in Pickering emulsions. Two-dimensional (2D) materials present a promising route toward nanosheet-stabilized emulsions for functional segregated networks, while also facilitating surface energy studies. Here, we demonstrate emulsions stabilized by the 2D materials including the transition metal carbide MXene, titanium carbide (Ti_3_C_2_T*_x_*), and develop an approach for *in situ* measurement of nanosheet surface energy based on emulsion inversion. This approach is applied to determine the influence of pH and nanosheet size on surface energy for MXene, graphene oxide, pristine graphene, and molybdenum disulfide. The surface energy values of hydrophilic Ti_3_C_2_T*_x_* and graphene oxide decrease significantly upon protonation of usually dissociated functional groups, facilitating emulsion stabilization. Similarly, pristine graphene and molybdenum disulfide increase in surface energy when their surface functional groups are deprotonated under basic conditions. In addition, the surface energies of these pristine materials are correlated with nanosheet size, which allows for the calculation of the basal plane and edge surface energies of pristine nanosheets. This understanding of surface energies and control of emulsion inversion will allow design of emulsion-templated structures and surface energy studies of a wide range of solution-processable nanomaterials.

## Introduction

Two-dimensional (2D) materials are an exciting class of materials where their range of properties can be realized in solution-processable nanosheets.^1^ However, there are still major challenges in the development of many applications that require controlled macroscopic assembly of 2D nanosheets.^2,3^ Specifically, their surface properties including surface energy, which governs interfacial assembly, are poorly understood and known to depend on a number of factors such as pH and nanosheet size.^4^ So far, a detailed study of the relationship between surface energy and dispersion properties has been difficult, as there are a limited number of methods for *in situ* measurement of the surface energy of dispersed nanosheets.^4^ In this work, we present a novel technique to obtain the surface energy values of dispersed 2D nanosheets and apply the method to a range of materials of different lateral sizes under different pH conditions.

**Figure 1:**
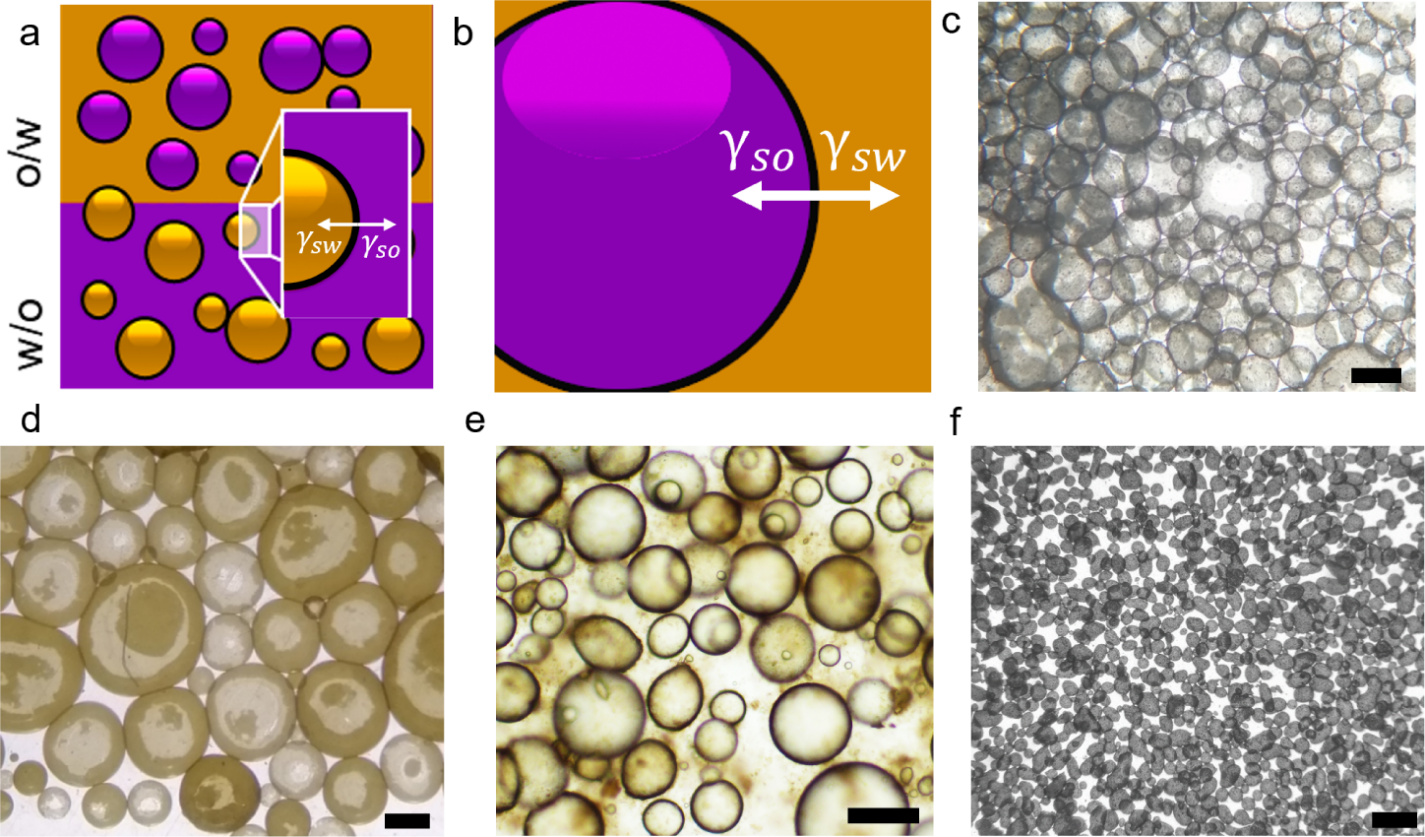
(a) Top: schematic of an oil-in-water (o/w) emulsion. Oil droplets were emulsified in a water matrix, with a nanosheet stabilizer at the interface between the phases. Bottom: schematic of a water in oil (w/o) emulsion. The orientation of the emulsion is dictated by the interfacial energies (γ_int_) between the solid and oil phase (γ_so_) and the solid and water phase (γ_sw_) which can be approximated as given in Supporting Information. The inset shows these forces acting at the three-phase boundary. (b) Enlarged graphic of the three-phase boundary and the forces acting on it, as in (a). Optical micrographs of emulsions prepared from water and cyclopentanone stabilized by different 2D materials. (c) Graphene (scale bar = 500 μm). (d) MoS_2_ (scale bar = 500 μm). (e) GO (scale bar = 200 μm). (f) Ti_3_C_3_T_*x*_ MXene (scale bar = 500 μm).

Recently, Pickering emulsification has been applied to 2D materials such as few-layer graphene^3,5−7^ as well as MoS_2_ and hexagonal boron nitride as a method of templated nanosheet network assembly. Pickering emulsions can be defined depending on the phase (i.e., water or oil) that forms the droplet and the matrix. An emulsion where the oil phase forms the droplets and the water phase forms the matrix is referred to as oil-in-water (o/w) as opposed to water-in-oil (w/o) with the oil phase in the droplets and the water phase in the matrix (Figure 1a). To illustrate the forces acting on the interface, Figure 1b shows an individual oil droplet in a water matrix, coated by a 2D material. In this system, the surface tension between the solid and the oil phase (Γ_so_) and that between the solid and the water phase (Γ_sw_) act perpendicular to the solid surface in opposing directions. Their relative magnitudes dictate the emulsion orientation, where the one with the larger surface tension with the solid stabilizer forms the matrix phase.

2D materials offer a range of functional properties and surface energies, which lead to different emulsification behaviors.^8^ Among those that can be emulsified in most water–oil systems, as they have similar γ_s_ of around 70 mJ/m^2^, are graphene, boron nitride, and transition metal dichalcogenides (TMDs), as shown previously.^1,9^ Most studies have been limited to w/o emulsions and those stabilized by poorly exfoliated graphene with correspondingly high loading levels.^2,3,6^ We have recently demonstrated several well-exfoliated and size-selected nanosheet-stabilized emulsions, which remain electrically conductive, even at ultralow loading levels to enable an array of applications, including sensing. In addition, we have demonstrated that emulsion orientation can be controlled by tuning the surface energy and pH,^7^ enabling the production of emulsion-templated silicone strain sensors.^2^

So far, tailoring the orientation of emulsions has proven to be a major challenge for developing applications and has also limited the choice of potential solid stabilizers, hindering the application of, for example, hydrophilic materials such as graphene oxide (GO) and MXenes.^10−12^ This is because their orientation depends on the stabilizer surface energy and the surface energies of the liquid phases used.^13^ This work develops an understanding of the relationship between surface energy, pH, and particle size, providing a platform for fundamental surface studies and tailoring emulsion structure toward specific applications.

## Methods

Graphite (Kibaran Resources Limited) is an air-classified powder with a D90 of 50 μm. The surfactant Triton X-100, phosphoric acid, sulfuric acid, potassium hydroxide, and the solvents used for emulsification (*n*-pentane, cyclohexanone, cyclopentanone, dichloromethane, and ethyl acetate) were purchased from Merck. Using a Thermo Scientific Barnstead MicroPure system, deionized water (DI) with a resistivity of 18.2 MΩ·cm was prepared. The MoS_2_ powder was purchased from Sigma-Aldrich with a D90 of <2 μm, and the graphene oxide was purchased as an exfoliated stock dispersion from Graphenea. The Ti_3_C_2_T*_x_* MXene used in this work was synthesized as described by Maleski et al.^11^ Colloidal solutions of delaminated Ti_3_C_2_T_*x*_ material dispersed in deionized water and sealed in vials filled with argon. Organic solvent dye Oil Red Orange and water-soluble green fluorescent salt were purchased from Merck and used at concentrations of 0.1 g/L.

Bath sonication in neutral DI water for 30 min at 15 °C was used to aid the exfoliation of MXene. At a concentration of 6 g/L, the MXene was diluted with a phosphoric acid solution to yield a dispersion of controlled pH, as further detailed in Supporting Information. This step was repeated with the GO dispersion, which was diluted to a concentration of 1 g/L. A high-pressure homogenization process developed by Large et al. was used for the exfoliation of MoS_2_ and graphene.^14^ In this process, the graphite powder is added to a 4 g/L premixed solution of Triton X-100 in DI water at a mass content of 60 g/L. The dispersion is then homogenized in 0.5 L batches, using the optimized process parameters of 241 MPa operating pressure, 20 °C chiller temperature, and ∼16 recirculation passes. For MoS_2_, the approximate volume ratio of surfactant to crystallite was kept constant and the homogenization process was carried out in the same manner.

### Homogenization

Homogenization of surfactant dispersions was performed in a reverse flow configuration using a BEE International Mini DeBEE high-pressure homogenizer and diamond nozzle (∼100 μm aperture). The temperature was controlled using an Applied Thermal Control Ltd. K4 4.5 kW recirculating chiller, capable of controlling temperatures between 5 and 35 °C. During processing, the thermal set point of the system was maintained to a 0.5 °C tolerance.

### Size Selection

To select the different 2D materials by size, we used liquid cascade centrifugation (LCC), a liquid processing technique based on iterative centrifugation cascades which involves controlled sedimentation of nanosheets as proposed by Backes et al.^8^ Centrifugation of homogenized dispersions was performed using a Beckman Coulter Avanti J15-R benchtop centrifuge with a JS-4750 swinging bucket rotor with a maximum 3 L capacity (4 × 750 mL polypropylene centrifuge tubes) in the case of graphene and a Sorvall Legend X1 Centrifuge for MoS_2_. The centrifugation speed applied to each fraction is detailed in Supporting Information.

In subsequent detailed evaluations of the surface energy–size relationships, all LCC fractions, as detailed in Supporting Information, were measured and labeled with their respective centrifugation speed and time product. For the preliminary size-dependent surface energy experiments, two MoS_2_ samples and two graphene samples were selected. In the following, the sample centrifuged at a speed of 500 gmin is labeled large graphene (MLG, < L > = 840 nm), while the sample centrifuged at 32000 gmin is labeled small graphene (FLG < L > = 140 nm). For MoS_2_, the samples centrifuged at 15000 gmin and 60000 gmin were selected and labeled large MoS_2_ (ML MoS_2_, < L > = 390 nm) and small MoS_2_ (FL MoS_2_, < L > = 240 nm), respectively.

### Surface Tension Measurement of Liquids

Solvents and solvent mixture surface tensions were measured with the Wilhelmy plate method, using a NIMA Langmuir trough.

### Surface Energy Measurement of 2D Materials

**Figure 2:**
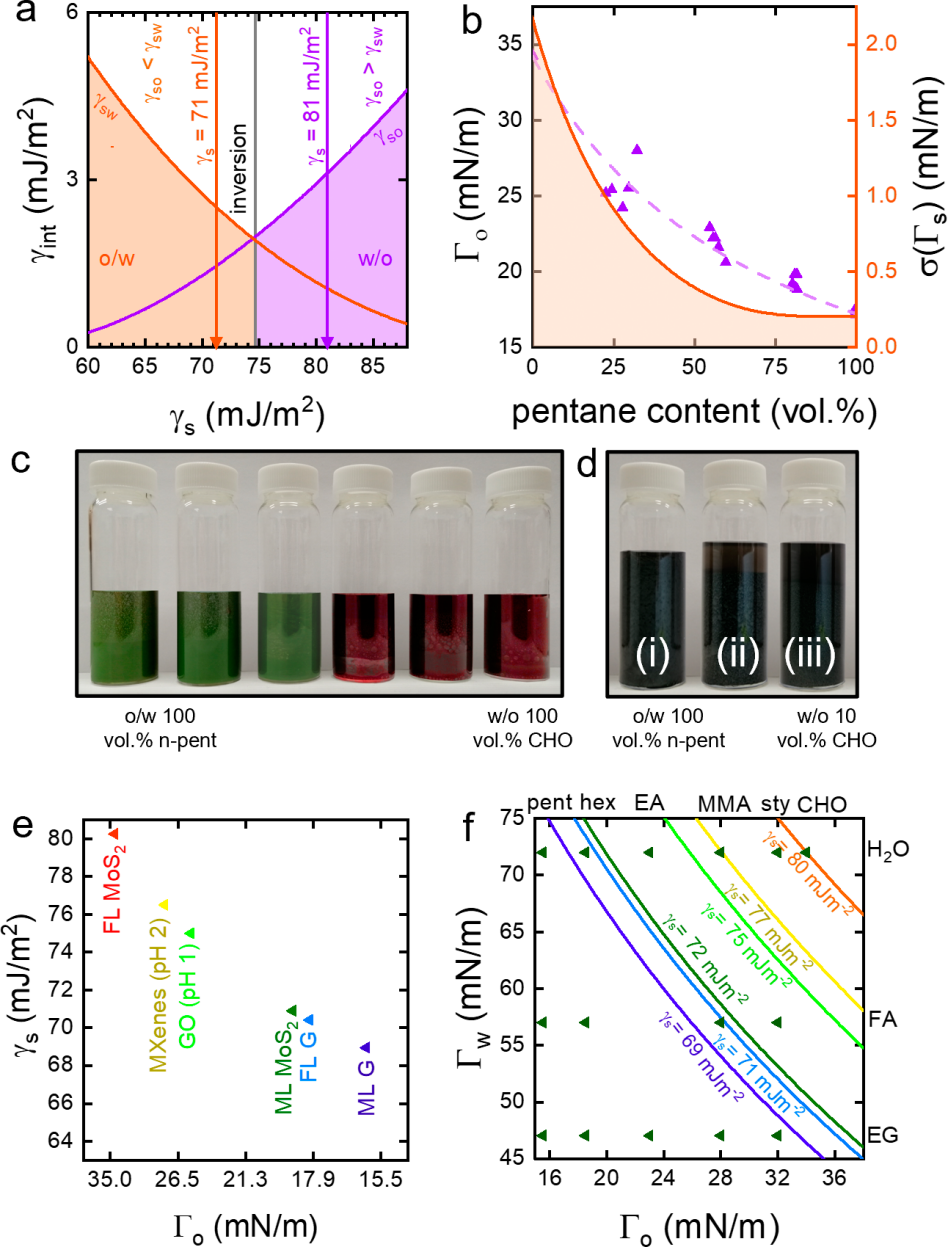
(a) Interfacial energy between emulsion components as a function of surface energy of the solid (γ_s_) shows regions of stable emulsion formation and points of emulsion inversion for ethyl acetate (EA) and water. (b) The surface tension of the oil phase as a function of *n*-pentane content in the *n*-pent/CHO mixture and corresponding uncertainly in the measured value of solid surface energy. (c) Photograph of the batch experiment to measure nanosheet surface energy with an organic phase of different compositions, colored with red dye and water colored with green dye. From 60 vol % to 70 vol % of *n*-pentane, an inversion from o/w to w/o is observed, clearly visible through a transition from buoyant oil droplets in the green water matrix to sedimented water droplets in the red oil matrix. (d) Photograph of the titration experiment used to determine γ_s_ showing o/w emulsion (i) and gradual destabilization on CHO addition to form a w/o emulsion (ii) which is stable after 10 s (iii). (e) The surface energies of different materials plotted against the surface tension of the oil phase at the inversion point between w/o and o/w. (f) The curves show the solutions of eq 1 for different materials (color scheme as in e). The surface tensions of different solvent combinations are plotted, including the largely water immiscible solvents *n*-pentane (pent), *n*-hexane (hex), EA, methyl methacrylate (MMA), styrene (sty), and cyclohexanone (CHO) and the largely water (H_2_O) miscible solvents formamide (FA) and ethylene glycol (EG). The combinations on the right side of a curve yield w/o emulsions for the regarded w/o emulsions, while those combinations on the left yield o/w emulsions.

The surface energies were determined using a phase inversion experiment, where the surface tension of the oil phase was gradually changed, while the water-miscible phase (composed of water or EG) surface tension would remain constant. For the size-dependent measurement of graphene, titration experiments in a water and CHO/*n*-pent system and an EG and DCM/hex system were used. For the size-dependent measurements of MoS_2_ and for all pH-dependent measurements, batch experiments with a step width of 10 vol % were used. For each composition, two vials were prepared with 10 mL of solvent each and 0.3–1.0 g/L 2D materials dispersion, where one of the vials remained untreated (hydrophilic surface) and one was coated with polydimethylsiloxane (hydrophobic surface). This helps to determine the orientation of the emulsion. The inversion point from o/w to w/o allows calculating the surface energy of the solid stabilizer according to eq 1, where  and  refer to the average between the surface tensions of the last stable o/w emulsion and the first stable w/o emulsion. The orientation was determined using a red organic solvent dye and a fluorescent salt in the emulsion system, so that the matrix phase color indicates the emulsion orientation, as can be seen in Figure 2c,d.

### Atomic Force Microscopy

AFM measurements were carried out using a Bruker Dimension Icon instrument with a ScanAsyst silicon nitride probe with a tip radius of 5–10 nm. Samples were scanned at areas of 5 × 5 μm^2^ and 10 × 10 μm^2^ at a resolution of 256/line at a scan rate of 0.5 Hz. The resulting images were analyzed by using NanoScope Analysis 2.0 software. Five profiles were taken of each sample in different locations, and nanosheet sizes of representative samples were analyzed. The AFM images and corresponding histograms can be found in Supporting Information.

### UV–Visible Spectrophotometry

Spectroscopy of dispersions was performed by using a Shimadzu UV3600 Plus UV–vis-NIR spectrometer. From these spectra, we determined the concentration, layer number, and lateral size of the dispersions with metrics established by Backes et al.^15^

### Zeta Potential

Zeta potential was measured by electrophoretic light scattering using an Anton Paar LiteSizer 500 and an Anton Paar Univette.

### Optical Microscopy

An Olympus BX53 M optical microscope with a 4K digital charge-coupled device camera was used to capture optical micrographs of emulsions.

### pH Measurement

The pH of the diluted samples was measured using a Thermo Scientific Orion Star A111 pH meter and Ag/AgCl electrode.

## Results and Discussion

Figures 1c–f show optical microscopy of emulsion droplets stabilized by nanosheets of (c) graphene, (d) MoS_2_, (e) GO, and (f) Ti_3_C_2_T_*x*_ MXene trapped at the interfaces between the phases. In order to emulsify MXene and GO, the dispersion was acidified to manipulate the surface functional groups.

The orientation is determined by the interfacial tensions between the liquid phases and the solid, γ_sw_ and γ_so_, as illustrated in Figure 2a. If γ_sw_ > γ_so_, an o/w emulsion will form, and *vice versa*, so a change in the liquid phase surface energy can result in a change of orientation. At the inversion point between w/o and o/w, the surface energies of the constituting liquid phases can be used to approximately measure γ_s_ of the nanosheet using eq 1 with γ_w,i_ and γ_o,i_ being the average liquid phase surface energies between the last o/w and the first w/o emulsion.
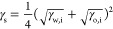
1

We have previously reported the use of phase inversion experiments for *in situ* measurements of nanomaterial’s surface energy, where we detailed the derivations used to obtain eq 1.^7^ This derivation can also be found in Supporting Information. Here, we have extended our work to distinguish differences and trends in the surface energy change of 2D materials and link these directly to changes in the surface chemistry. In the following, surface energy γ _s_ and surface tension Γ_s_ are used interchangeably, with the surface energy being the sum of the surface tension and the surface entropy. The surface entropy takes the universal value for surface entropy of about 0.1 mJ/m^2^K, which is ∼29 mJ/m^2^ at room temperature.^1^ It should be noted however that the distinction between surface energy and surface tension is not always acknowledged in the literature but is important for emulsions due to the mathematical form of eq 1.

Cycloketones, such as cyclohexanone (CHO) and cyclopentanone (CPO), allow exfoliation of bulk-layered materials to few-layer nanosheets, to maximize surface area and minimize loading of nanosheets, and facilitate emulsification with water.^7,17,18^ Their high surface energy, compared to other water-immiscible organics, results in a tendency of pristine 2D materials to form w/o emulsions, as γ_so_ will generally be higher than γ_sw_. To place bounds on γ_s_, the inversion point between the w/o and o/w emulsion must be determined, which can be achieved by decreasing the surface tension of the oil phase. The surface tension of either phase can be gradually changed by mixing a solvent of high surface tension and a solvent of low surface tension, as shown in Figure 2b. The component with the lower surface energy will enrich at the liquid–air interface; therefore, some deviation of the linear, ideal mixing behavior is observed. This is well-described by an established model developed by Backes et al., which is detailed in Supporting Information. The measured surface energies of different phase compositions were fitted with this model to allow prediction of the surface tension of variable phase compositions, as plotted in purple in Figure 2b (left axis) for a pentane-CHO mixture.

Solvent evaporation, miscibility, and accuracy of the Wilhelmy plate method affect the accuracy of γ_o_, while γ_w_ is mainly affected by miscibility. Gaussian error propagation is used to determine the resulting uncertainties of the phases’ surface tensions, σ(γ_o_) and σ(γ_w_), as detailed in Supporting Information. The uncertainty σ(γ_s_) is plotted as a function of *n*-pentane content in Figure 2b in orange. A maximum uncertainty σ(γ_s_) of 2.1 mJ/m^2^ suggests that the measurement of the surface energy of 2D materials in this system is sufficiently robust to develop design rules for the controlled emulsion assembly.

Understanding the factors that influence γ_s_ allows for control of the orientation and stability of emulsions to allow, for example, droplet deposition or composite formation. For this reason, the γ_s_ measurement approach was applied to several aqueous dispersions of 2D materials obtained by exfoliation in the presence of a surfactant, followed by washing using centrifugation. Triton X-100 was used as a surfactant as it improves exfoliation results while not influencing the orientation or stability of the emulsion.^7^ Working with an aqueous phase, however, limits the ability to obtain o/w emulsions with common organic solvents due to the high surface tension of the water. Phase inversion experiments to measure the surface energy can be realized using two methods; γ_s_ can be determined with a batch experiment, as shown for MoS_2_ in Figure 2c, or in a titration experiment, as shown for graphene in Figure 2d. In the batch experiment, different mixtures of varying vol % of *n*-pentane (and CHO) are prepared and their orientation is determined. Γ_o_ at the inversion points can then be calculated from the vol % of *n*-pentane near the orientation transition as shown in Figure 2b. In the titration experiment, γ_o_ is changed by the gradual addition of CHO to pentane. After crossing the inversion point, as illustrated in Figure 2a, the o/w emulsion in Figure 2d(i) collapses to form a w/o emulsion, shown after 10 s in (ii) and after 1 min in (iii).

The Γ_o_ values at the inversion points of different materials are plotted against the corresponding surface energy of the solid in Figure 2e, calculated from eq 1, with γ_w_ = 101 mJ/m^2^ (H_2_O). We evaluated dispersions of different materials obtained by liquid cascade centrifugation with respect to their surface energy. Selected samples are plotted in Figure 2e to provide a qualitative measure of the large differences in surface energy. For both graphene and MoS_2_, two different samples were selected, which differed in nanosheet size. The largest size fraction of graphene, referred to as MLG, has average lateral size < L > = 840 nm, while the smallest size fraction, FLG, has < L > = 140 nm. As for MoS_2_, the largest size fraction, referred to as ML MoS_2_, has < L > = 390 nm, while the smallest, FL MoS_2_, has < L > = 240 nm.

Measurement of γ_s_ for different materials allows to plot eq 1 as a phase diagram, given in Figure 2f, where (Γ_w_, Γ_o_) combinations to the right of a given inversion curve threshold yield w/o emulsions, whereas (Γ_w_, Γ_o_) combinations to the left lead to o/w emulsions. Figure 2f shows that an emulsion with methyl methacrylate (MMA) and water yields a w/o emulsion with small graphene, whereas H_2_O and pentane lead to an o/w emulsion with small graphene. This allows the design of emulsion structures depending on the stabilizer, properties of the liquid phases, pH, and nanosheet size.

**Figure 3:**
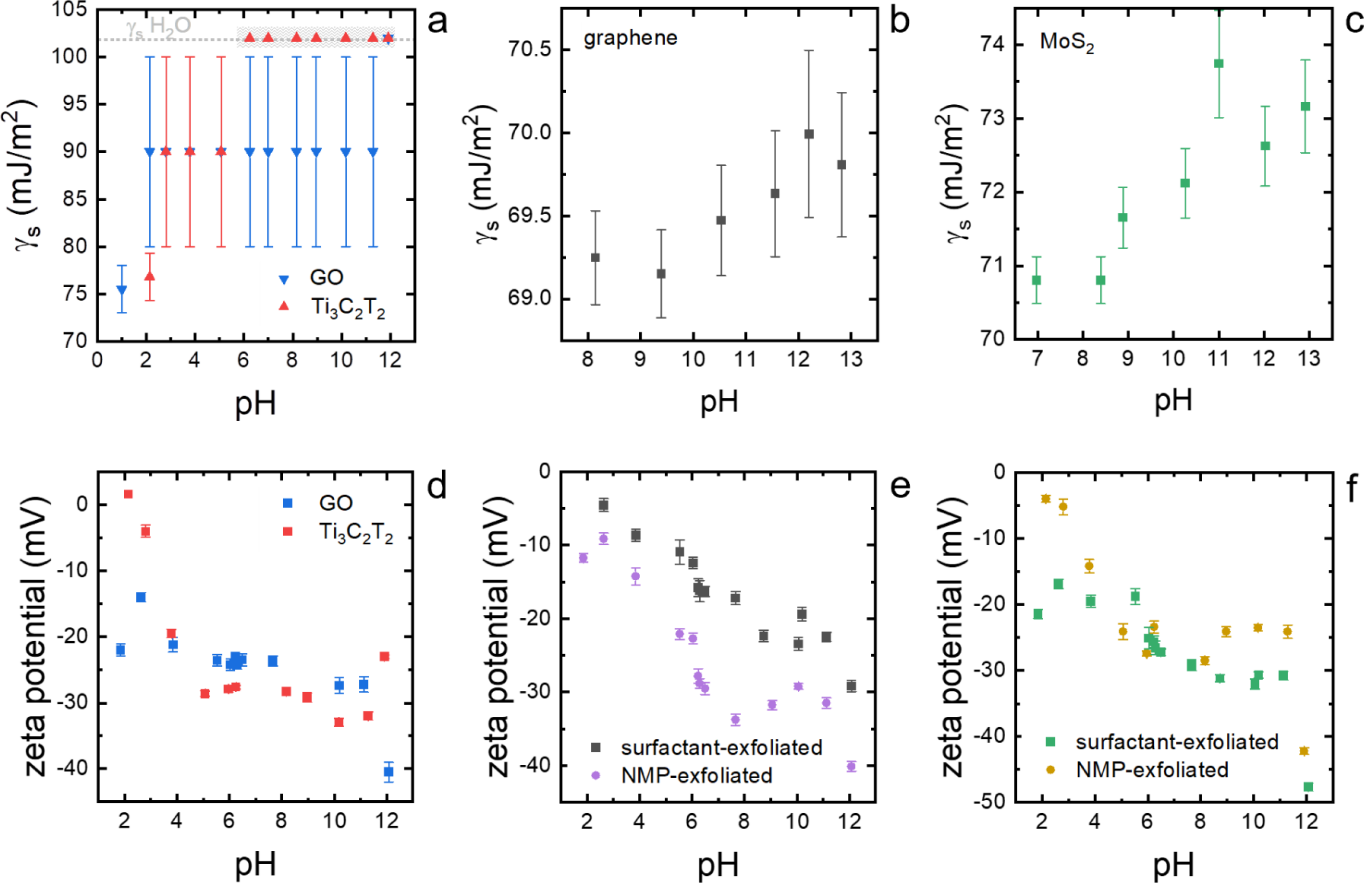
(a) Surface energy of GO and Ti_3_C_2_T_*x*_ as a function of pH showing threshold at pH 1.5 and 2.5 for Ti_3_C_2_T_*x*_ MXene and GO, respectively, where the surface energy drops below that of CHO, allowing the surface energy to be determined accurately by inversion of the emulsion. At higher pH, in the range from 2.5 to 5.5 for Ti_3_C_2_T_*x*_ MXene and 1.5–11.5 for GO, the materials can be emulsified but not inverted due to their higher surface energy, leading to large constant uncertainty in surface energy given by these bounds. Above these ranges, the materials do not assemble at the interface due to their high surface energy. (b) Surface energy of the surfactant-exfoliated graphene dispersion as a function of pH. (c) Surface energy of the surfactant-exfoliated MoS_2_ dispersion as a function of pH. (d) The zeta potential of GO and Ti_3_C_2_T_*x*_ MXene. (e) The zeta potential of graphene, exfoliated in a water–surfactant medium and an organic solvent medium. (f) The zeta potential of MoS_2_, again exfoliated in both water–surfactant and organic solvent, was also measured as a function of pH, showing the same trend as that of graphene.

Most hydrophilic materials such as GO, Ti_3_C_2_T*_x_* MXene, and other materials with high surface energy, such as particles with very small (<150 nm) lateral dimensions, cannot form emulsions above a certain pH. This is because they are not wetted by the oil phase if all surface functional groups are deprotonated, as S_so_ is positive. This contradicts the requirement of eq S1 and leads to the material not assembling at the interface at all. Decreasing the pH leads to a reduction of γ_so_ and γ_sw_ through protonation of dissociated functional groups on the surface of the 2D materials and leads to their trapping at the interface, as observed in GO by He et al.^19^ Reprotonation of the material’s functional groups results in a decrease of the polar contribution γ_s_^p^, resulting in decreased γ_sw_ and γ_so_, as can be derived from eq S6. Ti_3_C_2_T*_x_* MXene and GO dispersions were adjusted to pH 2.5 and pH 1.5, respectively, using sulfuric acid (H_2_SO_4_) and used immediately, to avoid any agglomeration, to prepare emulsions. Through emulsification, it is therefore possible to measure γ_s_ of the acidified material, where previously it was impossible to place bounds on γ_s_ of dispersed material. The dependence of surface energy on pH for MXene and GO is shown in Figure 3a. These measurements indicate that for basic and neutral pH, it is possible only to bound the surface energy by the (in)stability of the emulsions. When acidified to pH 2 for MXene or pH 1 for GO, an emulsion inversion allows the surface energy to be determined with values close to 75 mJ/m^2^ for both materials.

By extension, the surface energy of pristine (unfunctionalized, hydrophobic) 2D materials such as graphene and MoS_2_ can be manipulated by changing pH. This is explained by the edge functionalities, which deprotonate at high pH and raise the polar contribution γ_s_^p^ to γ_s_ and *vice versa* (see Supporting Information). Thus, o/w emulsions are formed even in systems of high γ such as H_2_O and CHO. The change in the surface energy of pristine graphene (< L > ∼ 500 nm) occurs gradually between pH 9 and 12, as shown in Figure 3b, and amounts to at least 1 mJ/m^2^, whereas that of MoS_2_ (< L > ∼ 500 nm) amounts to at least 3 mJ/m^2^ as shown in Figure 3c. As the influence of pH depends on the defect density, the changes in surface energy with pH increase as the material size decreases. Acidification in turn decreases the surface energy of pristine materials, which enables the formation of emulsions with very small few-layer graphene and MoS_2_ that would otherwise have too high surface energy to allow assembly at the interface.

Zeta potential measurements were taken to determine the charge state of the surface. They were used to understand changes in the surface energy of GO, Ti_3_C_2_T*_x_* MXene, graphene, and MoS_2_ dispersions in terms of changes of their surface charge as shown in Figures 3d–f. Generally, there is a strong correlation between the zeta potential change and the change in surface energies with pH, confirming the hypothesis that the change in surface energy is a result of changing surface functional group protonation. GO shows almost constant zeta potential across the pH spectrum except for an increase at pH 2 and a strong decrease below pH 11. Ti_3_C_2_T_*x*_ MXene however only shows a strong decrease in zeta potential between pH 2 and 5, and zeta potential remains constant at higher pH, in agreement with a previous study on the zeta potential response to pH of Ti_3_C_2_T_*x*_.^20^ The zeta potential of graphene shows a gradual decrease with increasing pH, changing slightly depending on whether a surfactant (TX-100) is present in the system. While TX-100 seems to influence the overall zeta potential, the trend remains unchanged. The zeta potential of MoS_2_ was also measured as a function of pH, showing the same trend as graphene. Thus, zeta potential measurements can be used to predict the sensitivity of surface energy to pH changes for preliminary experiments, allowing screening to be carried out with less material than the actual surface energy measurements.

This pH dependence of the surface energy allows the formation of emulsions of 2D materials with water and various organic phases. This is particularly relevant for polymer composites, since many organics such as styrene or MMA can be polymerized to have relatively high surface tensions and thus form w/o emulsions with water.^21^ Due to the pH dependence of the surface energy, however, the orientation of the emulsion can be tailored even with phases composed of solvents with high surface tension.

**Figure 4:**
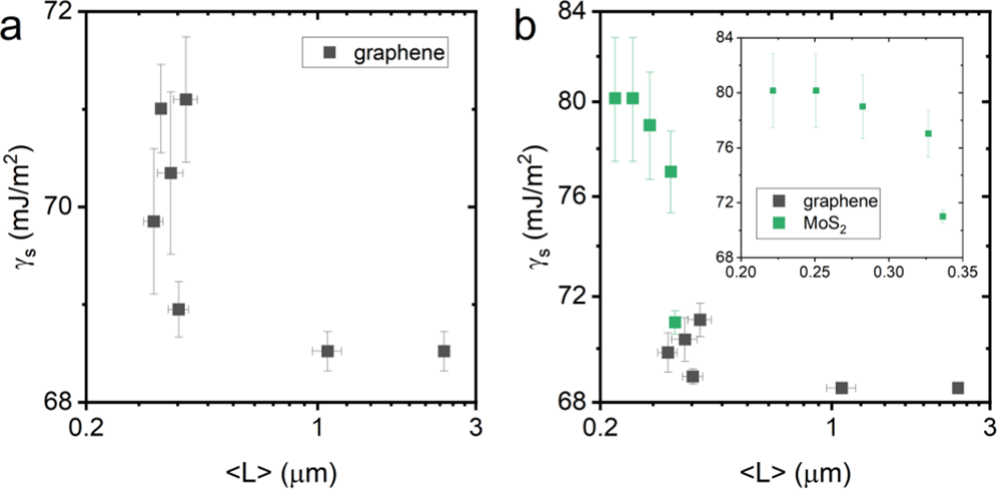
(a) Surface energy of size-selected dispersions of graphene. (b) Surface energy of size-selected dispersions of MoS_2_.

In addition, the influence of nanosheet size on surface energy was studied, as smaller sheets are known to have a higher surface energy than larger sheets.^4^ This is to be expected, as small sheets have a higher density of high-energy edge defects. The metastable singlet radical bonds in pristine graphene edge sites react and form functional groups such as hydroxyl groups in the presence of reactants.^22−24^ This increases the polar contribution to the surface energy, as described in eq S6. Graphene and MoS_2_ were size-selected using liquid cascade centrifugation, and their surface energies were measured using a phase inversion batch experiment. Their surface energy changes significantly with the average sheet length for both materials, as can be seen in Figure 4a,b for MoS_2_ and for graphene. This allows further tuning of the orientation of emulsions for specific applications.

Ferguson et al.^4^ conducted surface energy studies of graphite and graphene using inverse gas chromatography. Through modeling of the data, they could differentiate the graphene basal plane surface energy contributions (γ_s,b_) from the graphene edge (γ_s,ed_) and basal plane defect surface energy (γ_s,bd_) contribution. They found γ_s,b_ to be ∼61 mJ/m^2^, γ_s,ed_ to be ∼130 mJ/m^2^, and γ_s,bd_ to be ∼180 mJ/m^2^. To obtain an estimate for the basal plane surface energy of graphene using a phase inversion method, the water phase was substituted with ethylene glycol (EG). With a surface energy of 47.3 mN/m, which is much lower compared to that of water (77.0 mN/m), EG enables the measurement of much lower surface energies with a phase inversion experiment. We measured the graphene dispersion in EG with very large nanosheet size (< L > ∼ 840 nm) which yielded a basal plane surface energy γ_s,b_ ∼ 61 mJ/m^2^, which is in excellent agreement with the results of Ferguson et al.^4^

Subsequently, size selection cascades of graphene and MoS_2_ were measured in a water-CHO/pentane phase system, yielding the surface energies shown in Figure 4. Both graphene and MoS_2_ show the expected increase in surface energy with reducing lateral size, indicating a high surface energy contribution from defects at edge sites. The surface energy for large graphene nanosheets shown in Figure 4a tends toward ∼68 mJ/m^2^ and increases relatively sharply to ∼71 mJ/m^2^ for smaller nanosheets. For MoS_2_, the range of accessible lateral sizes limited by small bulk powder size yields higher surface energies and limits the sizes at which the emulsion orientation can be inverted to study this size dependence. As shown in Figure 4b, the largest MoS_2_ fractions are determined to have surface energies ∼71 mJ/m^2^, comparable to the smallest graphene fractions, while the smaller MoS_2_ fractions, like graphene, show a sharp increase to >77 mJ/m^2^. It is interesting to note that while the size and surface energy ranges for graphene and MoS_2_ are nearly distinct, their values form a continuous data set, potentially indicating similar surface energies of basal plane and size-dependent edge contributions, although both exhibit sharp increases which are not described by simple geometric models. This clear reduction in surface energy with nanosheet size presents an additional route to tuning emulsion orientation through control of nanosheet properties to enable assembly of a range of functional structures.

## Conclusion

Nanosheet-stabilized emulsions offer a route toward templated assembly of 2D materials, allowing the retention of their high specific surface area. While MoS_2_, graphene, and GO emulsions have previously been demonstrated, here we present that Ti_3_C_2_T*_x_* MXene, despite its high hydrophilicity, can be emulsified with various organic solvents under acidic conditions. Furthermore, this work introduces an approach to accurately measuring the surface energy of dispersed nanomaterials using a phase inversion experiment. The method can be applied to a range of materials, including graphene and MoS_2_, to determine processing-based variations in surface energy for control of subsequent applications. The ability to measure the surface energy of 2D materials *in situ* provides detailed insights into how pH influences the surface energy of 2D materials. Changes in surface energy induced by protonation or deprotonation of the functional groups and defects on the materials allow tailoring the emulsion orientation for applications including sensors and energy storage. Given that defect density is related to nanosheet size, surface energy increases with decreasing average lateral size of the nanosheets in the dispersion as demonstrated for pristine graphene and MoS_2_, providing a facile route toward design of emulsion structures. This approach will facilitate fundamental surface energy studies of a wide range of nanomaterials and conditions, elucidating new understanding through simple interfacial assembly.

## Supporting Information Available

Surface energy measurement approach; size selection; size- and pH-dependent surface energy measurements (PDF)
