# Supplementary material for: Measuring the Surface Energy of Nanosheets by Emulsion Inversion
Source: J Phys Chem C Nanomater Interfaces. 2024 Oct 1;128(40):17073–80. doi: 10.1021/acs.jpcc.4c02893 (PMC11474924; doi:10.1021/acs.jpcc.4c02893)
Supplement: Supplementary file 1 — jp4c02893_si_001.pdf [file jp4c02893_si_001.pdf]

Supporting Information for

Measuring the Surface Energy of Nanosheets by Emulsion Inversion

Anne Sehnal[1], Sean P. Ogilvie[1]*, Keiran Clifford[1], Hannah J. Wood[1], Aline Amorim Graf[1], Frank Lee[1], Manoj Tripathi[1], Peter J. Lynch[1], Matthew J. Large[1], Shayan Seyedin[2], Kathleen Maleski[3], Yury Gogotsi[3] and Alan B. Dalton[1]*

[1]School of Mathematical and Physical Sciences, University of Sussex, Brighton, BN1 1RH, UK

[2]School of Engineering, Newcastle University, Newcastle upon Tyne, NE1 7RU, UK

[3]A. J. Drexel Nanomaterials Institute, and Department of Materials Science and Engineering,

Drexel University, Philadelphia, PA 19104, USA

* s.ogilvie@sussex.ac.uk; a.b.dalton@sussex.ac.uk

Surface energy measurement approach

The formation of stable Pickering emulsion depends on the wettability of the solid, which is quantitatively described in equations S1 and S2, where S_{so} is the oil on the solid and S_{sw} is the water on the solid. Furthermore, the solid needs to have a positive energy of adhesion to the liquid phases, as expressed in equations S3 and S4.[1]

The matrix phase of the emulsion can either be constituted of the oil phase (red) with the aqueous phase forming the droplets (water in oil, w/o), or vice versa (oil in water, o/w). The former orientation is schematically shown in figure 1 a (i), while the latter can be seen in figure 1 a (ii). The spreading coefficient also determines the orientation of the emulsion. The preferentially wetting liquid phase, with larger spreading coefficient S will constitute the matrix phase, while the other phase will form the droplets. Hence, when $S_{so} > S_{sw}$ a w/o emulsion is formed, while when $S_{sw} > S_{so}$ an o/w emulsion is formed. [1]

$$S_{so} = \gamma_{so} - \gamma_{sw} - \gamma_{ow} < 0 \quad \text{(S1)}$$

$$S_{sw} = -\gamma_{so} + \gamma_{sw} - \gamma_{ow} < 0 \quad \text{(S2)}$$

$$E_{adh,so} = \gamma_{so} - \gamma_{sw} + \gamma_{ow} > 0 \quad \text{(S3)}$$

$$E_{adh,sw} = -\gamma_{so} + \gamma_{sw} + \gamma_{ow} > 0 \quad \text{(S4)}$$

With γ_{ow} in equations S1 to S2 constant, the interfacial tensions γ_{sw} and γ_{so} determine the relative magnitude of S and hence the orientation of the emulsion, as illustrated in figure 1 b. This can be approximated using the geometric mean from the bulk attractive forces in each phase, $\sqrt{\gamma_a\gamma_b}$, and the attractive forces operating across the interface itself, γ_a and γ_b as given in equation S5.[2]

$$\gamma_{ab} = \gamma_a + \gamma_b - 2\sqrt{\gamma_a\gamma_b} \quad \text{(S5)}$$

A more general model was developed by Fowkes[3] and extended by Owens and Wendt[4]. Their model differentiates the contributions of dispersive and polar intermolecular forces between the phases a and b to the surface tension, marked by d and p respectively, given in equation S6.

$$\gamma_{ab} = \gamma_a^d + \gamma_a^p + \gamma_b^d + \gamma_b^p - 2\sqrt{\gamma_a^d \gamma_b^d} - 2\sqrt{\gamma_a^p \gamma_b^p} \quad \text{(S6)}$$

Substituting equation S1 and S2 with equation S5, we can derive an orientation criterion for the emulsions, given in equations S7 depending on the surface tensions of the constituting phases.

$$\text{o/w:}\ \ \gamma_s > \frac{1}{4}\left(\sqrt{\gamma_w} + \sqrt{\gamma_o}\right)^2 \qquad \text{w/o:}\ \ \gamma_s < \frac{1}{4}\left(\sqrt{\gamma_w} + \sqrt{\gamma_o}\right)^2 \quad \text{(S7)}$$

Binary mixing

Using a binary mixture as the oil phase allows gradual change of γ_o that can be calculated using the simple correlation in equation S8, established by Eberhart et al.[5] It depends on the volume fractions (φ) of the solvents a (lower γ) and b (higher γ), with $\varphi_b = 1 - \varphi_a$, γ the surface energy of the pure components (γ) and S, an empirical solvent-mixture specific parameter determined through calibration. S = 1 for ideal mixtures with homogenous distribution of a and b, S > 1 for surface enrichment in the lower γ component a.

$$\gamma_{o(\varphi_a)} = \frac{S\,\varphi_a \gamma_a - (1 - \varphi)\gamma_b}{S\varphi_a + (1 - \varphi_a)} \ \ \text{with}\ \ \varphi_b = 1 - \varphi_a \quad \text{(S8)}$$

Robustness evaluation

Using a binary mixture as the oil phase allows gradual change of γ_o.[5] V represents the volume fractions of the solvents a (lower γ) and b (higher γ), with $V_b = 1 - V_a$, γ the surface tension of the pure components and S an empirical solvent-mixture specific parameter determined through calibration. S = 1 for ideal mixtures with homogenous distribution of a and b, S > 1 for surface enrichment in the lower SE component a.

$$\gamma o(V_a) = \frac{S\,V_a \gamma_a + (1 - V_a)\gamma_b}{SV_a + (1 - V_a)} \ \ \text{with}\ \ V_b = 1 - V_a \quad \text{(S9)}$$

Evaporation of n-pentane during the calibration leads to some scatter in the data in figure 2 c for high pentane contents resulting in an uncertainty of the parameter S of 8.2%. One further source of uncertainty are the surface tensions of the pure components, CHO γ_b and n-pentane γ_a, due to the accuracy of the microbalance and the Wilhelmy plate method. No change in the surface tension of pure organic components due to miscibility effects could be measured. The measurements of pure solvents showed very small standard deviations of 0.05 mNm^{-1} for CHO and 0.17 mNm^{-1} for n-pentane. This suggests that the method is very accurate, but there is a possibility of systematic errors possibly due to errors in the size of the plate, that cannot be accounted for here. The surface tension of n-pentane was measured as 17.1±0.17 mNm^{-1} and that of CHO as 34.2 ± 0.05 mNm^{-1}. While n-pentane has a negligible miscibility with water, CHO dissolves in water up to 86 gL^{-1}. Miscibility does not affect the surface tensions of the pure organic phases, the surface tension of the mixed phases however is altered, due to some of the CHO dissolving in the water phase. This leads to generally lower surface tensions in the organic phase when in contact with water and is here accounted for as an uncertainty in the CHO content (V_b) of 8.6 V%.

Using Gaussian error propagation, the error of the calculated value for γo can be calculated, as follows:

$$\sigma(\gamma o) = \sqrt{\left(\frac{\partial\gamma o}{\partial S}\cdot\sigma(S)\right)^2 + \left(\frac{\partial\gamma o}{\partial\gamma a}\cdot\sigma(\gamma a)\right)^2 + \left(\frac{\partial\gamma o}{\partial\gamma b}\cdot\sigma(\gamma b)\right)^2 + \left(\frac{\partial\gamma o}{\partial Vb}\cdot\sigma(Vb)\right)^2} =$$

$$= \sqrt{\left(\frac{(Va - Va^2)(A - B)}{(SVa + 1 - Va)^2}\sigma(S)\right)^2 + \left(SVa\,\sigma(\gamma a)\right)^2 + \left((1 - Va)\,\sigma(\gamma b)\right)^2 + \left(\frac{-S(A + B)}{(SVa + 1 - Va)^2}\sigma(Vb)\right)^2}$$

With:

$\sigma(S)$… Uncertainty in S, $\sigma(S)$= 0.19

$\sigma(\gamma a)$… Uncertainty in γ_a, $\sigma(\gamma_a)$= 0.17 mNm^{-1}

$\sigma(\gamma b)$… Uncertainty in γ_b, $\sigma(\gamma_b)$= 0.05 mNm^{-1}

$\sigma(Vb)$… Uncertainty in Vb, $\sigma(Vb)$= 0.09 Vb, if step width between fractions is considered, $\sigma(Vb)$= 0.09 Vb+0.1

The surface tension of graphene is calculated from the surface tensions $\gamma o \pm \sigma(\gamma o)$ and $\gamma w \pm \sigma(\gamma w)$ at the inversion point according to equation S5, with $\sigma(\gamma w)$ estimated to be 1.5 mNm^{-1}, due to miscibility.

$$\sigma(\gamma s) = \sqrt{\left(\frac{\sigma(\gamma_w)}{\sqrt{\gamma_w}}\right)^2 + \left(\frac{\sigma(\gamma_o)}{\sqrt{\gamma_o}}\right)^2} \qquad (S10)$$

Substituting this equation allows to plot the uncertainty $\sigma(\gamma_s)$ as a function of n-pentane content, as shown in figure 2 c.

With a maximum error of 2.1 mNm^{-1}, it can be said that the measurement of the surface energy of graphene is very robust against miscibility, evaporation and scatter in the acquired data, assuming an accurate measurement of the surface tension with the Wilhelmy plate method. In a batch experiment, the step width between the samples must be considered, which leads to slightly higher uncertainties depending on the chosen experiment. The robustness of this method is further supported by the agreement of our results with values obtained using Inverse Gas Chromatography by Ferguson et al.[6]

Size selection

The graphene and MoS_2 dispersions were size selected using a liquid cascade centrifugation method as first introduced by Backes et al.[7] The process involves iterative centrifugation cascades, where the sediment of a centrifugation step is collected and labelled with the product of the centrifugation time and the relative centrifugal field (RCF*t). Subsequently, the supernatant is centrifuged again at a higher relative centrifugal force and with increased centrifugation time. The sediment of the subsequent step is collected again and the supernatant subjected to further steps of centrifugation.

MoS_2

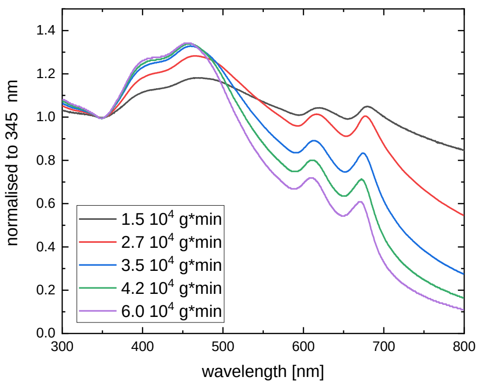

Figure S1: UV-visible spectra of different LCC size fractions.

Graphene

The surface energies of graphene of different sizes, obtained from liquid cascade centrifugation, were determined using a titration experiment with graphene dispersed in water and an oil phase constituted of different ratios of CHO and n-pentane.

γ_o is changed by gradual addition of CHO to pentane. It is critical to simultaneously add an equivalent amount of water phase to the system, so that the phase volume ratio of the immiscible phases remains constant. The step width when sweeping g_o must be considered when evaluating the accuracy of γ_s, as larger steps in γ_o lead to larger uncertainties in γ_s.

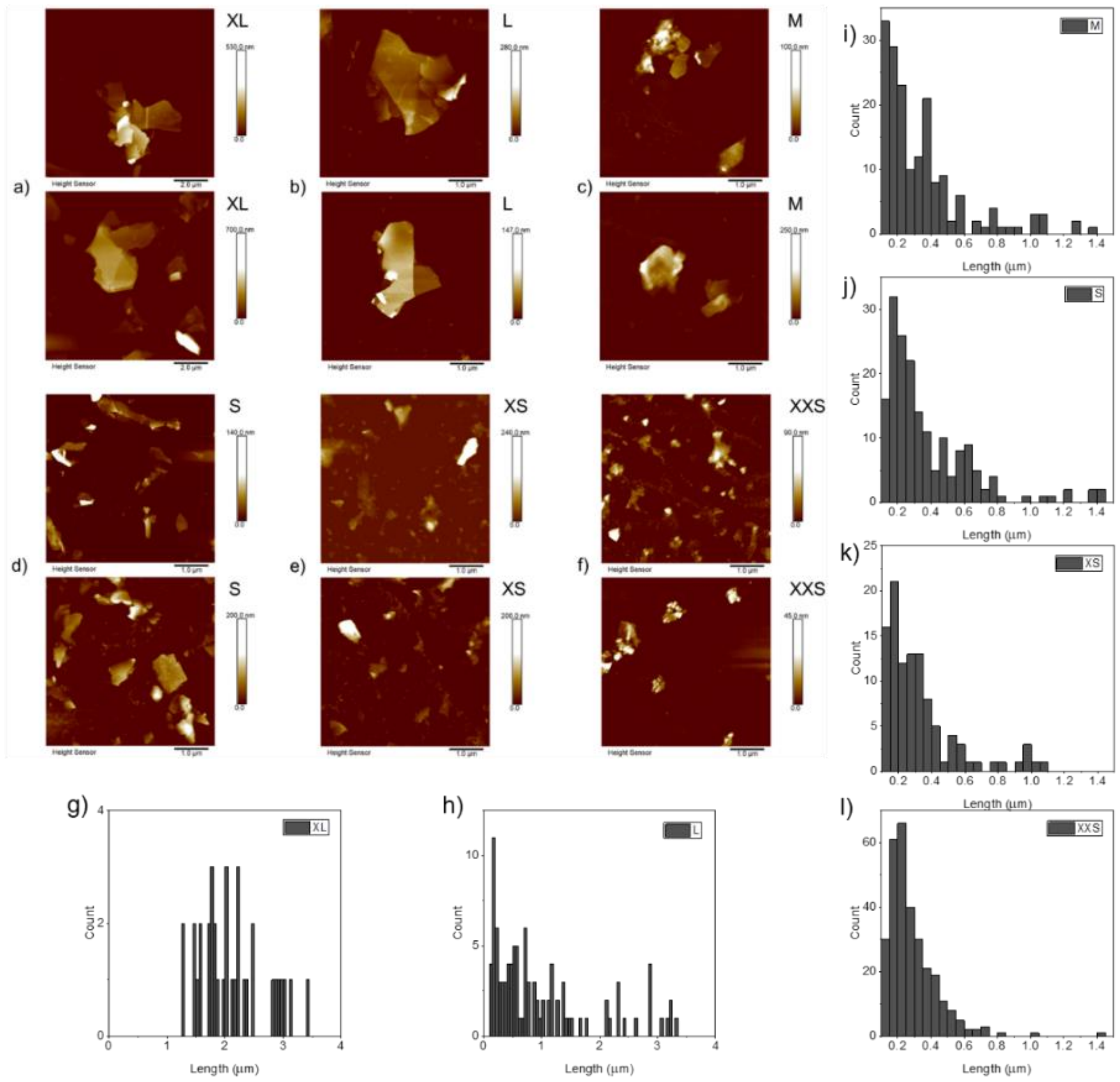

Figure S2: Representative AFM of the graphene size fractions and corresponding size distribution histograms.

Size- and pH-dependent surface energy measurements

MoS_2

The surface energies of MoS_2 obtained from liquid cascade centrifugation were measured with an oil phase composed of CHO and n-pent and water. The inversion points are determined by calculating the average of the phase composition of the first o/w emulsion and the last w/o emulsion. From about 100 nm lateral size of the MoS2 flakes on, it was not possible any more to determine the surface energy of the flakes with a phase inversion experiment, as the material, due to its high surface energy, only forms o/w emulsions in liquid systems commonly accessible.

pH-dependent measurements

The MXenes and GO dispersions were acidified in a controlled manner to determine the re-protonation pH of the different materials. To obtain a MXenes dispersion of pH 5, pH4 phosphoric acid solution, neutral MXenes and GO dispersions and neutral DI water were mixed with 1:1:8 ratio. The same procedure was applied to obtain pH 4, pH3 and pH2 dispersions. This way, accurate pH could be achieved without requiring measurement of the pH of the acidified dispersion every time.

The pH 1 dispersions were obtained by mixing stock phosphoric acid solution (pH 1) with aqueous MXenes and GO dispersions at 9:1 ratio, resulting in a real pH of the dispersion slightly above 1.

After successfully emulsifying the MXenes at pH 1 and pH 2, the surface tension was determined with a water-CPO-EA phase inversion experiment. The inversion was observed between 80 V% CPO to 20V% EA (w/o) and 60 V% CPO and 40 V% EA (o/w). After shaking the vials, the emulsion at pH 1 collapsed due to the phosphoric acid dissolving in the organic phase and protonating CPO, leading to increased miscibility of the phases. The pH 2 emulsion remained intact after shaking.

GO was successfully emulsified at pH 1 and subsequently the surface tension was determined using an EA DCM mixture as oil phase while not altering the aqueous phase. The inversion of the orientation from o/w to w/o could be observed between 75 V% DCM and 25 V% EA and 100 V% DCM as oil phase.

References

1. Chevalier, Y. & Bolzinger, M.-A. Emulsions stabilized with solid nanoparticles: Pickering emulsions. *Colloids Surf. Physicochem. Eng. Asp.* **439**, 23–34 (2013).
2. Girifalco, L. A. & Good, R. J. A Theory for the Estimation of Surface and Interfacial Energies. I. Derivation and Application to Interfacial Tension. *J. Phys. Chem.* **61**, 904–909 (1957).
3. Fowkes, F. M. DETERMINATION OF INTERFACIAL TENSIONS, CONTACT ANGLES, AND DISPERSION FORCES IN SURFACES BY ASSUMING ADDITIVITY OF INTERMOLECULAR INTERACTIONS IN SURFACES. *J. Phys. Chem.* **66**, 382–382 (1962).
4. Owens, D. K. & Wendt, R. C. Estimation of the surface free energy of polymers. *J. Appl. Polym. Sci.* **13**, 1741–1747 (1969).
5. Eberhart, J. G. The Surface Tension of Binary Liquid Mixtures [1]. *J. Phys. Chem.* **70**, 1183–1186 (1966).
6. Ferguson, A., Caffrey, I. T., Backes, C., Coleman, J. N. & Bergin, S. D. Differentiating Defect and Basal Plane Contributions to the Surface Energy of Graphite Using Inverse Gas Chromatography. *Chem. Mater.* **28**, 6355–6366 (2016).
7. Backes, C. *et al.* Production of Highly Monolayer Enriched Dispersions of Liquid-Exfoliated Nanosheets by Liquid Cascade Centrifugation. *ACS Nano* **10**, 1589–1601 (2016).
